# Osteogenesis ability of CAD/CAM porous zirconia scaffolds enriched with nano-hydroxyapatite particles

**DOI:** 10.1186/s40729-017-0082-6

**Published:** 2017-05-19

**Authors:** Moustafa N. Aboushelib, Rehab Shawky

**Affiliations:** 1 Dental Biomaterials Department, Faculty of Dentistry, Alexandria University, Champollion st, Azarita, Alexandria, Egypt; 2 Oral Surgery Department, Faculty of Dentistry, Alexandria University, Alexandria, Egypt

**Keywords:** Nano-porous, Hydroxyapatite coating, Zirconia, Scaffold

## Abstract

**Background:**

The aim of this study was to evaluate osteogenesis ability of CAD/CAM porous zirconia scaffolds enriched with hydroxy apatite used to augment large boney defects in a dog model.

**Methods:**

Surgical defects were made bilaterally on the lower jaw of 12 Beagle dogs. Cone beam CT images were used to create three dimensional images of the healed defects. Porous zirconia scaffolds were fabricated by milling custom made CAD/CAM blocks into the desired shape. After sintering, the pores of half of the scaffolds were filled with a nano-hydroxy apatite (HA) powder while the other half served as control. The scaffolds were inserted bilaterally in the healed mandibular jaw defects and were secured in position by resorbable fixation screws. After a healing time of 6 weeks, bone-scaffold interface was subjected to histomorphometric analysis to detect the amount of new bone formation. Stained histological sections were analyzed using a computer software (*n*=12, *α*=0.05). Mercury porosimetery was used to measure pore sizes, chemical composition was analyzed using energy dispersive x-ray analysis (EDX), and the crystal structure was identified using x-ray diffraction micro-analysis (XRD).

**Results:**

HA enriched zirconia scaffolds revealed significantly higher volume of new bone formation (33% ± 14) compared to the controls (21% ± 11). New bone deposition started by coating the pore cavity walls and proceeded by filling the entire pore volume. Bone in-growth started from the surface of the scaffold and propagated towards the scaffold core. Islands of entrapped hydroxy apatite particles were observed in mineralized bone matrix.

**Conclusions:**

Within the limitations of this study, hydroxy apatite enhanced osteogenesis ability of porous zirconia scaffolds.

## Background

Principles of tissue engineering are used today in an attempt to reconstruct damaged human tissue. In the dental field, several types of bone grafting materials are currently available which could be directly used to augment atrophic jaws before implant placement. However, the main drawback of these grafts is related to difficulty of preserving the required shape of the graft during the healing time [1]. Protecting the grafting material using titanium meshes or other temporary devices may not be applicable in non-accessible areas. Nevertheless, using autogenous bone blocks may not be an acceptable option for many patients due to the expected morbidity of the donor site [2].

Today, different types of pre-shaped bone grafting materials are currently available for augmenting atrophic ridges. One of the greatest challenges facing successful ridge augmentation is to maintain the desired shape after soft tissue closure [3]. Several studies reported a high rate of bone resorption after insertion of implants in bone augmented ridges [4, 5]. On the other hand, porous non resorbable scaffolds have the advantage of maintaining ridge shape and dimensions during healing time.

Yttrium partially stabilized tetragonal zirconia polycrystalline material, known commercially in the dental field as zirconia, was first used in orthopedic surgery as a hip joint prosthesis [6]. Soon the material gained diverse applications in the dental field as a core and framework material, implant abutment, implant fixture, and a bone scaffold as well. Using computer-assisted design and milling technology (CAD/CAM), the fabrication of accurate and precise restorations became a simple procedure [3, 7].

One of the desired features of a bone scaffold is to resist functional loads as well [8]. Several studies evaluated bone reaction against optimized zirconia scaffold surfaces [9]. Studies ranging from cell culture to full scale animal models have indicated that osteointegration observed on optimized zirconia surfaces was equal if not superior to different materials, namely titanium alloy. These studies either focused on optimizing the surface structure of zirconia scaffolds controlling its porosity, geometric structure, and micro-roughness, or by coating the surface with a bioactive layer to enhance the process of osteogenesis ability [10–13]. Porous zirconia scaffolds could also be used as a drug delivery vehicle to enhance bone response as well [14]. A recent study attributed enhanced cell viability to the internal structure of the scaffold rather than to the type of coating material used [15].

Modern radiographic imaging techniques in combination with advanced computer designing software could reconstruct a three-dimensional image of large boney defects [16]. Designing the shape of the required scaffold could easily be performed to accurately fit the available defect size using advanced imaging and designing software [17]. Finally, the required shape could be directly milled from different materials using CAD/CAM technology and the milled scaffold could be inserted in the exposed boney defect.

The aim of this study was to evaluate osteogenesis ability of customized CAD/CAM porous zirconia scaffolds inserted in healed boney defects in the mandible of a dog model. The proposed hypothesis of this study was that hydroxyapatite-enriched zirconia scaffolds would enhance osteogenesis ability compared to the controls.

## Methods

### Preparation of the scaffold

Zirconia powder (50 μm, 3 mol YTZP, E grade, Tosoh, Japan) was mixed with 50 wt.% resin beads (50 μm polymethyl methacrylate powder) added to create microscopic pore sizes. Thirty weight percent coarse sodium chloride particles (500–700 μm) were added to the mix to create large interconnected pores. The powder was mixed in a rotating cylinder for 24 h to insure homogenous powder distribution. A binder material (1 wt.% polyvinyl glycol) was added to the mix, and the powder was isostatically pressed into cylinders (40 mm in diameter and 80 mm in length) to create the required dimensions of the CAD/CAM milling blocks. The blocks were partially sintered at 1250 ^○^C for 30 min then soaked in deionizer water for 24 h to dissolve the salt particles.

### Jaw defect and scaffold design

The ethics committee of Alexandria University approved the working protocol used in this study (STDF reintegration grant 489) according to the university code of conduct regarding using animals in scientific studies. After approval of the ethics committee on publishing parts of the obtained data, 2-year-old healthy male Beagle dogs (weighing 10–12 kg) were generally anesthetized by administration of subcutaneous injection of atropine (0.05 mg/kg; Kwangmyung Pharmaceutical, Seoul, Korea) and an intravenous injection of a mixed xylazine (Rompun, Bayer Korea, Seoul, Korea) and zoletil (Virbac, Carros, France) and maintained by inhalation anesthesia (Gerolan, Choongwae Pharmaceutical, Seoul, Korea). Surgical flap was reflected to expose the premolar-molar region of the lower jaw with minimum amount of trauma, afterwards the involved teeth were removed and a surgical block 2 cm long × 2 cm deep was cut using a surgical guide to demarcate the wound boundaries. Finally, the surgical flap was repositioned and sutured and the dogs received an antibiotic (20 mg/kg of cefazoline sodium, intramuscularly; Yuhan, Seoul, Korea) for 3 days, given soft diet, and the surgical site was sprayed with topical 0.2% chlorhexidine solution. After 3-month healing time, three-dimensional images of the boney defect was performed using cone beam CT radiographic imaging (I cat, Imaging science international, Hatfield, PA). The images were transferred to an open access CAD/CAM software (CAMworks, Geometric Americas INC, Scottsdale, AZ) and the design of the required zirconia scaffold was reconstructed to accurately fit the modeled boney defect putting in account the expected sintering shrinkage of the material (25 vol.% shrinkage). The scaffold was designed to restore normal contour of the resected ridge. Five axes dry milling unit (DWX-51D 5, Roland, Parkway, Irvin, Cal) was used to mill the prepared blocks into the required shape and the scaffold was sintered at 1350 ^○^C for 4 h.

### Enriching with nano-hydroxyapatite

Nano-hydroxyapatite particles were prepared using sol gel chemical precipitation method. The sol was thermally aged at low temperature at 50 °C for 2 h. Upon drying the sol particles agglomerated into a dry gel through van der Waals forces composed of 10–14-nm particles. A crystalline apatite is achieved after sintering at 450 °C resulting in a gained structure of 25–55 nm in diameter. Twenty-five weight percent suspension of nano-hydroxyapatite particles were added to 80% ethyl alcohol and stirred to achieve a homogenous suspension, and the right scaffold of each dog was immersed in the prepared suspension for 15 min under vacuum to insure adequate filling of all pores. Scaffolds were dried at 120 °C for 180 min and the process was repeated two times. Finally, the coated scaffolds were heated at 900 °C for 30 min to ensure proper drying of the particles without changing the chemistry of the particles or the supporting scaffold.

### Characterization of the prepared scaffolds

Mercury porosimetery was performed for evaluate pore size and distribution and to measure the total porosity percent of the scaffolds. Pore sizer (Porosimeter, Micromeritics 9320, USA) was used for testing the produced porosity on the nanoscale covering pore diameter in range from 360 to 0.006 μm.

Energy dispersive X-ray analysis (EDX) (INCA Penta FETX3, OXFORD Instruments, Model 6583, England) and X-ray diffraction analysis (XRD) (PANalytical, X Pert PRO, The Netherlands) with Cu target (*λ* = 1.54 Å), 45 kV, 40 mA, and 2*Ɵ* (10°–80°) were used to analyze elemental surface composition and crystal structure of the scaffolds. Density of the prepared scaffolds was compared to theoretical density of fully sintered zirconia.

### Surgical phase

Twelve weeks after healing of the resected ridges, the animals were exposed to the second stage surgery where the created defect size was exposed using the same procedures described previously and each scaffold was seated in its final position. Resorbable collagen membrane (Biomend, Zimmer Inc, CA, USA) was used to cover the exposed surface of the scaffold and soft tissue was gently expanded and sutured to secure proper wound closure using resorbable suture material (Vicryl Rapide 5; Ethicon Inc., Somerville, NY). To increase primary retention, the scaffolds were fixed using resorbable polylactic acid fixation screws (Rapidsorb, Deput Synthes, PA, USA).

### Histomorphometric analysis

Six weeks after insertion of the scaffolds, the animals were given an over dose of an anesthetic injection and section blocks were obtained by cutting the mandible maintaining 10 mm of sound bone around the scaffolds. Cut sections were immediately fixed in 4% buffered formaldehyde and dehydrated in graded ethanol solutions using a dehydration system under agitation and vacuum, and the specimens were then defatted in xylene solution. Finally, the specimens were embedded in transparent chemically polymerized methyl methacrylate resin (methyl methacrylate 99%, Sigma-Aldrich, Steinheim, Germany). After polymerization, the specimens were cut along the long axis of the scaffolds using a diamond-coated disc rotating in a micro-sectioning system (Micracut 150 precision cutter, Metkon, Bursa, Turkey) followed by polishing using 800 grit silicon carbide paper. One hundred-micrometer-thick cut sections were stained using Stevenel’s blue and van Gieson picro-fuchsin. Histomorphometric analysis was performed using digital images obtained using a light stereomicroscope (Olympus BX 61, Hamburg, Germany) equipped with a high-resolution digital camera (E330, Olympus, Imaging Corp, Beijing, China).

Measurements were made by first calculating the pore volume on the images using digital tracing option of the software (white pores on the images), and the amount of new bone formation (mineralized tissue stained red) was calculated as a percent of the total pore volume (Olympus CellM & CellR, version 3.3, Olympus Soft Imaging Solutions).

Examiner reliability was cross checked by re-evaluating randomly selected digital images by another expert examiner. The recorded correlation coefficient ranged from 0.83 to 0.92, indicating high reliability for all measured parameters. The data obtained were expressed as mean and standard deviation values and were analyzed using Student’s *t* test (SPSS 15.0, SPSS, Chicago, IL).

## Results

**Fig. 1:**
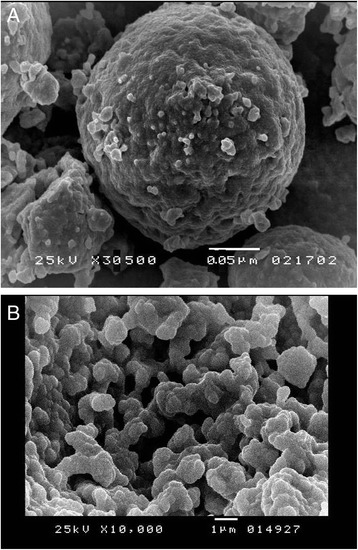
**a** SEM image, ×10,000, demonstrating internal porosity of the fabricated zirconia scaffolds. **b** SEM image, ×30,500, demonstrating agglomeration of nano-hydroxyapatite particles filling the porous structure

Mercury porosimetery revealed comparable (*F* = 0.057, *P* < 0.92) average pore diameter (85 ± 24 μm) for all the prepared scaffolds. Smallest pore diameter was 34 ± 2 μm and the largest pore diameter was 720 ± 13 μm. After filling the pores with hydroxyapatite, there was a significant (*F =* 16.1, *P <* 0.01) reduction in total porosity percent from 83 to 44 wt.% indicating that the nano-particles filled almost half of the available pores. There was also a significant reduction in average pore diameter from 85 ± 24 to 46 ± 29 μm. Amount of measured porosity was directly related to the measured bulk density of the scaffold structure. Agglomerates of hydroxyapatite particles were observed filling the porous structure (Fig. 1a, b).

EDX analysis of the enriched scaffolds revealed that Ca/P ratio was 1.67 indicating the presence of pure hydroxyapatite in the enriched scaffolds. XRD pattern revealed the characteristic peaks specific for the hexagonal HA crystal phase represented by (211), (112), and (300) peaks. Peaks of tetragonal yttrium zirconium oxide were detected for all specimens.

**Fig. 2:**
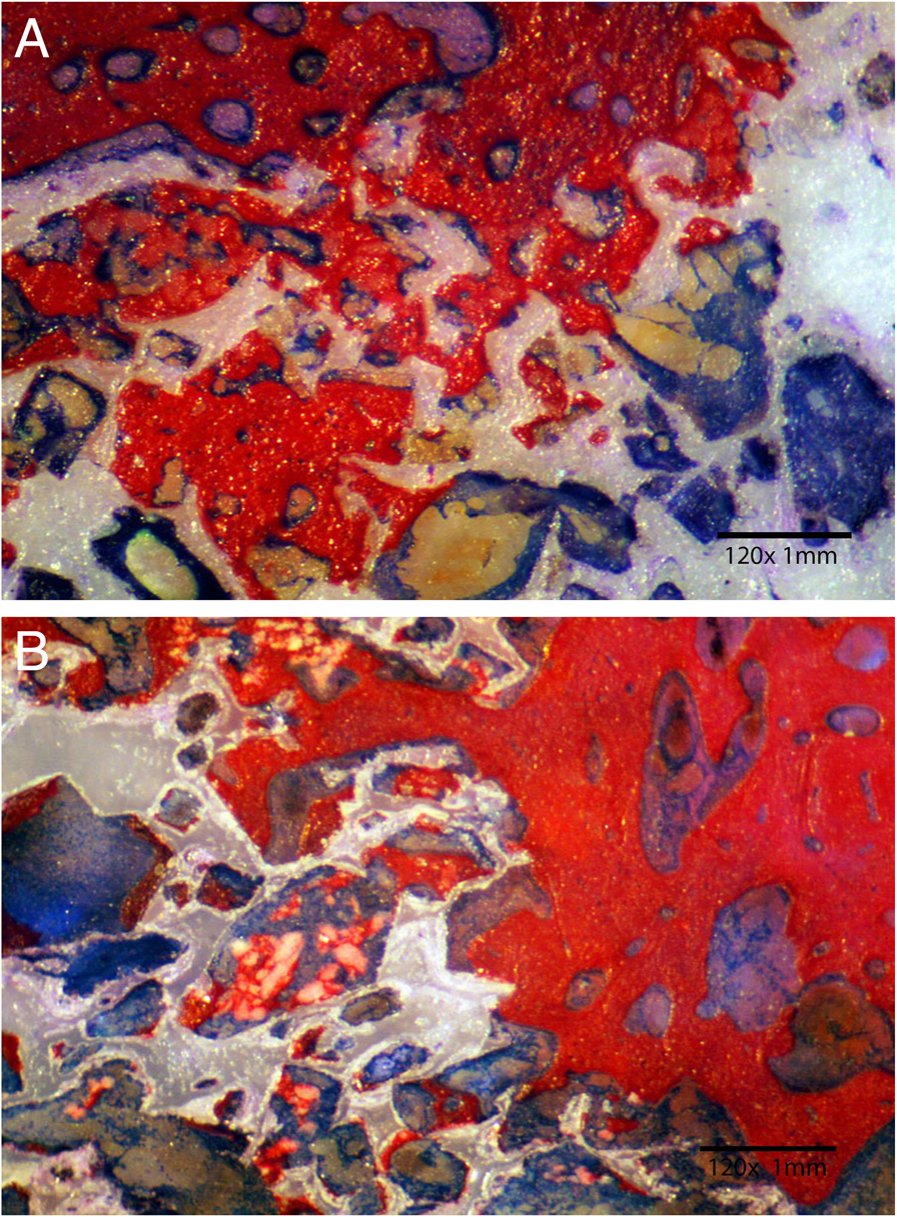
**a** Histological section demonstrating new bone growth (*white arrow*) in HA-enriched zirconia scaffold (*black arrow*). Unmineralized bone stained blue. Almost entire surface porosity was filled with new dense bone. **b** Histological section demonstrating bone growth in HA-enriched zirconia scaffold starting from the periphery of the surgical wound (*white arrow*). Islands of entrapped HA particles were surrounded by mineralized boney matrix (*black arrow*) which were identified using EDX

**Fig. 3:**
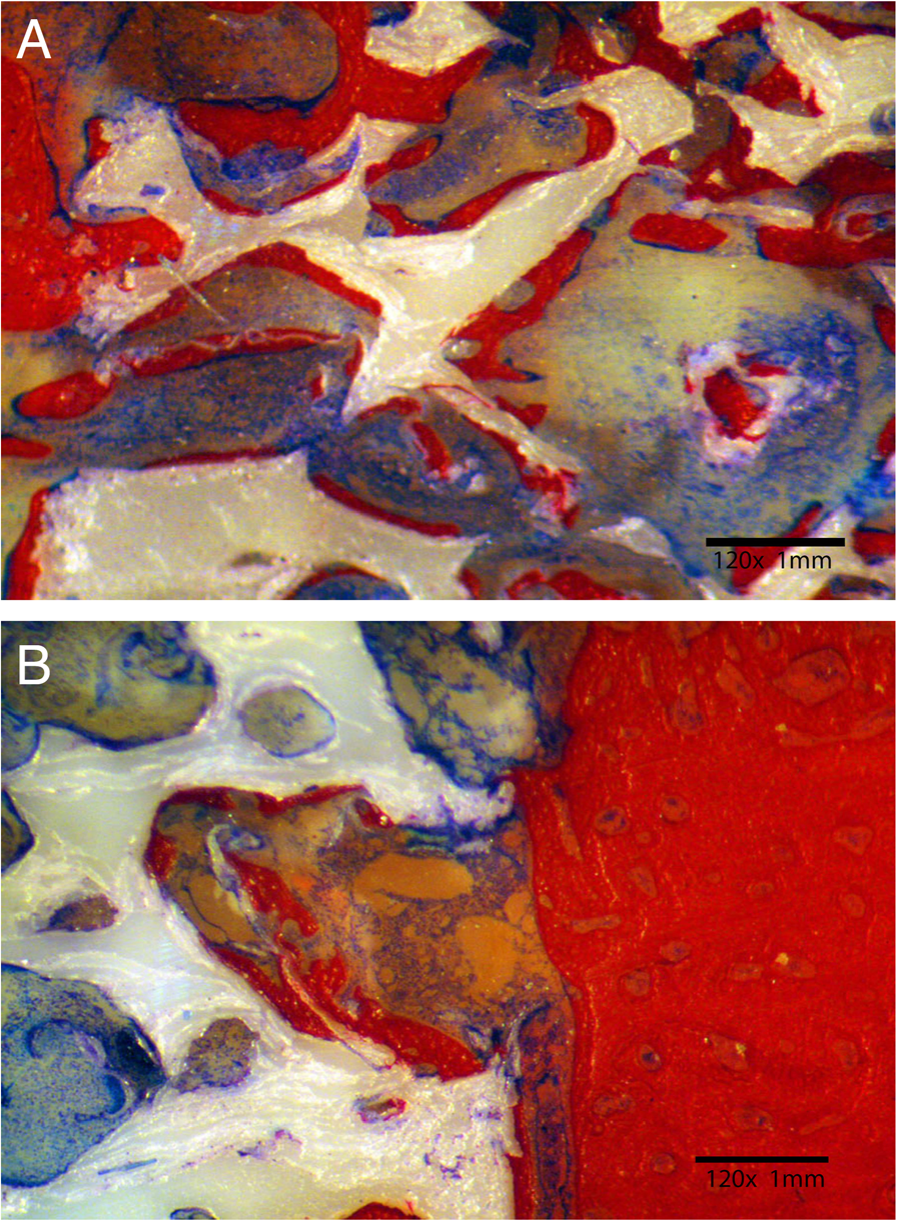
**a** Histological section demonstrating bone growth in control zirconia scaffold (*white arrow*). Mineralized bone formation (*black arrow*) was less dense compared to HA-enriched scaffolds. **b** Histological section showing different sizes of pores present in porous zirconia scaffolds (Control specimen). Mineralization started by lining pore walls (*white arrow*). Unmineralized bone stained blue

Histomorphometric analysis revealed that hydroxyapatite-enriched scaffolds had significantly (*F =* 14, *P <* 0.02) higher amount of new bone formation (33% ± 14) compared to the controls (21% ± 11). Amount of new bone formation was calculated as a percent of the total pore volume measured on each image. New bone growth started by lining pore cavity and propagated to gradually fill the entire pore volume (Fig. 2a). Bone ingrowth proceeded from the periphery of the scaffold and propagated towards its center (Fig. 2b). The surface under the guided tissue membrane was filled with unmineralized connective tissue. Regional areas of entrapped hydroxyapatite were observed inside the pore cavity of the enriched scaffolds (Fig. 2b). Entrapped islands of hydroxyapatite were surrounded by mineralized tissue. Lower amount of mineralized bone was observed for uncoated scaffolds (Fig. 3a, b).

**Fig. 4:**
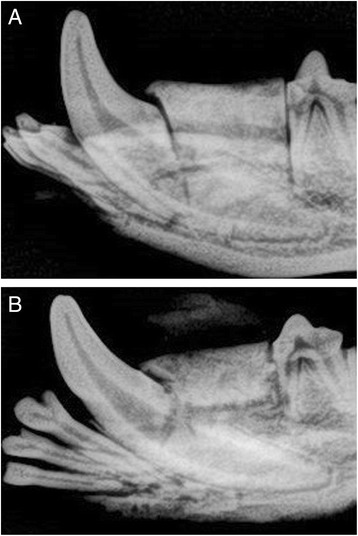
**a** Peri-apical X-ray of zirconia scaffold immediately placed in bone defect. Margins between scaffold and bone are clearly demarcated. **b** Peri-apical X-ray of zirconia scaffold after completion of healing time. Margins between bone defect and scaffold are less demarcated due to new bone growth

Radiographic examination revealed clear margins separating newly inserted scaffolds from surrounding bone defect (Fig. 4a). After completion of healing time, the margins between the scaffold and bone defect became less demarcated due to deposition and ingrowth of new bone (Fig. 4b).

## Discussion

Porous scaffolds are designed to allow ingrowth of the surrounding bone within the internal porosity of the solid matrix. Different types of bioactive materials were mixed with zirconia to enhance bone formation. Two sizes of pores were incorporated in the structure of the fabricated scaffolds. Micro-pores in range of 50 μm constituted the majority of the entire pore volume (50 wt.%) of the fabricated scaffolds (Fig. 1a). This pore volume was expected to provide adequate housing for osteoblasts and provide a mechanism of cell attachment on a microscopic level. Larger pore sizes (500–700 μm) reduced the density (30 wt.%) of the prepared scaffolds and created internal channels that connected different surfaces together creating a pathway for blood circulation [12, 15].

Previous research studies concerned with osteointegration of zirconia material reported comparable if not superior performance compared to different types of titanium alloys. This behavior was attributed to several factors related to the mechanical, physical, and chemical properties of zirconia [18]. With optimization of surface structure and geometry using a different technique, zirconia became available today as one piece dental implant [19].

Reconstruction of atrophic alveolar ridges could be achieved using a wide variety of grafting materials. However, maintaining the required shape of the graft represents a great challenge for the surgeon especially in regions subjected to functional loads. In the field of maxillofacial and cosmetic surgery, the graft must be mechanically strong to maintain its shape when placed in the surgical site and it must resist resorption and degradation to prevent collapse of the supported tissue [11].

Customized CAD/CAM porous zirconia scaffolds could easily be fabricated with high precision to fit the demands of the required surgical site. It could be used to augment atrophic alveolar ridges, replace bone loss in the maxillofacial region, and in cosmetic surgery as well. The scaffold is designed to be osteointgrated with the surrounding boney tissue thus it could perform mechanical function as well. The internal design of the scaffold enhanced blood circulation to ensure that the structure of the scaffold does not interrupt the biology of the surrounding tissue [15]. In combination with optimized nano-porous surface produced by selective infiltration etching, the scaffold could be enriched with different bioactive agents to enhance healing and osteogenesis ability with the surrounded tissue.

Histomorphometric analysis revealed that bone growth start to develop as early as 6 weeks by lining pore cavity walls. Mineralized bone matrix was observed to penetrate 1–2 mm under the surface of the scaffolds thus providing mechanical stability of the inserted prosthesis. Healing continued by filling the entire pore volume (Fig. 2a, b). The presence of nano-hydroxyapatite particles enhanced bone growth and deposition compared to uncoated surfaces (Fig. 3a, b). Hydroxyapatite enhanced osteogenesis ability of zirconia scaffolds, and the proposed hypothesis was accepted.

Kim et al. used coated zirconia scaffolds to augment calvarial defects in a rabbit model and reported closely matching values regarding porosity and density of the prepared scaffolds and regarding the amount of newly measured bone formation [20]. However, in this study, hydroxyapatite particles were not fused to the structure of the scaffold but were used to fill the pores resulting in much quicker release once in contract with body fluid which explains the superior performance of the enriched scaffolds.

Islands of hydroxyapatite particles were observed entrapped in the mineralized bone lining the internal pores of the enriched scaffold. Similar observation regarding the solubility of hydroxyapatite was reported in a cell culture study on porous zirconia surfaces [21]. In a clinical study, micro-porous-coated zirconia scaffolds demonstrated four times higher bone ingrowth and seven times higher bone-scaffold contact compared to uncoated scaffolds inserted in the maxilla of human patient [5].

Several trials used porous titanium scaffolds as a matrix for repair of large boney defects. Enriching these scaffolds with various growth factors enhanced clinical outcome [22–24]. However, the design of these scaffolds remains basically a network of interconnected wire structure that acts as a matrix filling the boney defect. CAD/CAM technology allows fabrication of custom-made zirconia scaffolds with full control over the distribution, size, and percentage of the created porosity.

In this study, the scaffolds were inserted in healed wounds in the alveolar ridge of dogs. The external surface of the scaffolds was protected by a resorbable-guided tissue membrane to prevent soft tissue migration which could compete with deposition of the required boney matrix. Traces of the collagen matrix of the membrane were observed on the free surface of the scaffolds. Being CAD/CAM fabricated from special milling blocks, the design of the scaffold could easily be tailored to meet the demands of different fields. In the field of implant dentistry, the future site of dental implant could be considered in the design of the scaffold to facilitate easier placement which will be considered in further studies.

## Conclusions

Within the limitations of this study, hydroxyapatite enhanced osteogenesis ability of porous zirconia scaffolds.
